# Spontaneous splenic rupture associated with scrub typhus: a case report

**DOI:** 10.1186/s40249-024-01177-5

**Published:** 2024-01-22

**Authors:** Hong Pil Hwang, Kyoung Min Kim, Hyojin Han, Jeong-Hwan Hwang

**Affiliations:** 1 Department of Surgery, Jeonbuk National University Hospital, Jeonbuk National University Medical School, Jeonju, Republic of Korea; 2 Department of Pathology, Jeonbuk National University Hospital, Jeonbuk National University Medical School, Jeonju, Republic of Korea; 3 Department of Internal Medicine, Jeonbuk National University Hospital, Jeonbuk National University Medical School, 20 Geonji-Ro, Deokjin-Gu, Jeonju-Si, Jeollabuk-Do 54907 Republic of Korea; 4 Research Institute of Clinical Medicine of Jeonbuk National University, Jeonju, Jeonjuk Korea; 5 Biomedical Research Institute of Jeonbuk National University Hospital, Jeonju, Jeonbuk Korea

**Keywords:** Scrub typhus, Complication, Splenic rupture

## Abstract

**Background:**

Scrub typhus, an acute febrile disease with mild to severe, life-threatening manifestations, potentially presents with a variety of complications, including pneumonia, acute respiratory distress syndrome, cardiac arrhythmias (such as atrial fibrillation), myocarditis, shock, peptic ulcer, gastrointestinal bleeding, meningitis, encephalitis, and renal failure. Of the various complications associated with scrub typhus, splenic rupture has rarely been reported, and its mechanisms are unknown. This study reports a case of scrub typhus-related spontaneous splenic rupture and identifies possible mechanisms through the gross and histopathologic findings.

**Case presentation:**

A 78-year-old man presented to our emergency room with a 5-day history of fever and skin rash. On physical examination, eschar was observed on the left upper abdominal quadrant. The abdomen was not tender, and there was no history of trauma. The *Orientia tsutsugamushi* antibody titer using the indirect immunofluorescent antibody test was 1:640. On Day 6 of hospitalization, he complained of sudden-onset left upper abdominal quadrant pain and showed mental changes. His vital signs were a blood pressure of 70/40 mmHg, a heart rate pf 140 beats per min, and a respiratory rate of 20 breaths per min, with a temperature of 36.8 °C. There were no signs of gastrointestinal bleeding, such as hematemesis, melena, or hematochezia. Grey Turner's sign was suspected during an abdominal examination. Portable ultrasonography showed retroperitoneal bleeding, so an emergency exploratory laparotomy was performed, leading to a diagnosis of hemoperitoneum due to splenic rupture and a splenectomy. The patient had been taking oral doxycycline (100 mg twice daily) for 6 days; after surgery, this was discontinued, and intravenous azithromycin (500 mg daily) was administered. No arrhythmia associated with azithromycin was observed. However, renal failure with hemodialysis, persistent hyperbilirubinemia, and multiorgan failure occurred. The patient did not recover and died on the fifty-sixth day of hospitalization.

**Conclusions:**

Clinicians should consider the possibility of splenic rupture in patients with scrub typhus who display sudden-onset abdominal pain and unstable vital signs. In addition, splenic capsular rupture and extra-capsular hemorrhage are thought to be caused by splenomegaly and capsular distention resulting from red blood cell congestion in the red pulp destroying the splenic sinus.

**Graphical abstract:**

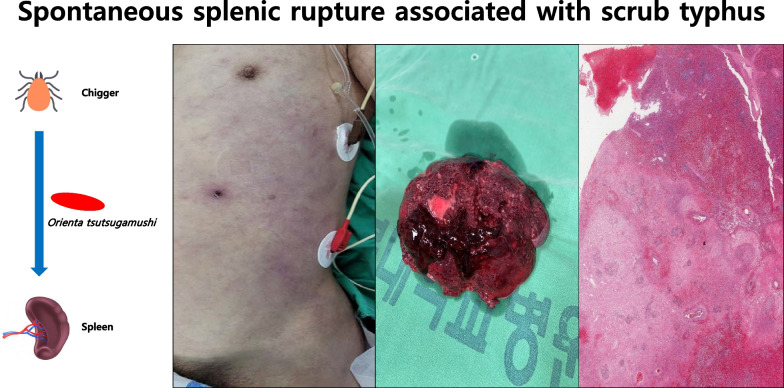

## Background

Scrub typhus is an acute, febrile, infectious disease caused by the obligate intracellular bacillus *Orientia tsutsugamushi* [1]. It is transmitted via the bites of chigger mites and leads to generalized or localized vasculitis that can involve any organ(s) [2]. Scrub typhus is a serious public health problem in the Asia–Pacific region, threatening a billion people globally, and there are a million cases each year [3]. Its manifestations range from mild to severe and life-threatening, and can be accompanied by various complications, such as pneumonia, acute respiratory distress syndrome, cardiac arrhythmias (such as atrial fibrillation), myocarditis, shock, peptic ulcer, gastrointestinal bleeding, meningitis, encephalitis, and renal failure [2]. The lungs are one of the main target organs for *O. tsutsugamushi*, leading to pulmonary complications of variable severity, and interstitial pneumonia may occur [1, 4]. Meningoencephalitis or encephalopathy can develop, resulting in agitation, delirium, or even have seizures [5]. Myocarditis can lead to cardiogenic shock, and while cardiac arrest has rarely been reported, recently the incidence seems to be increasing [6].

Among the various scrub typhus-related complications, splenic involvement, such as splenic rupture or splenic infarction, has been reported as rare [7]. Two cases of splenic rupture associated with scrub typhus have been reported in MEDLINE, 1 in PubMed and 1 in KoreaMed [8, 9]. The mechanism of scrub typhus-related splenic rupture is unknown. This study reports a case of spontaneous splenic rupture associated with scrub typhus in the Republic of Korea and identifies the possible mechanisms through the gross and histopathologic findings.

## Case presentation

**Fig. 1:**
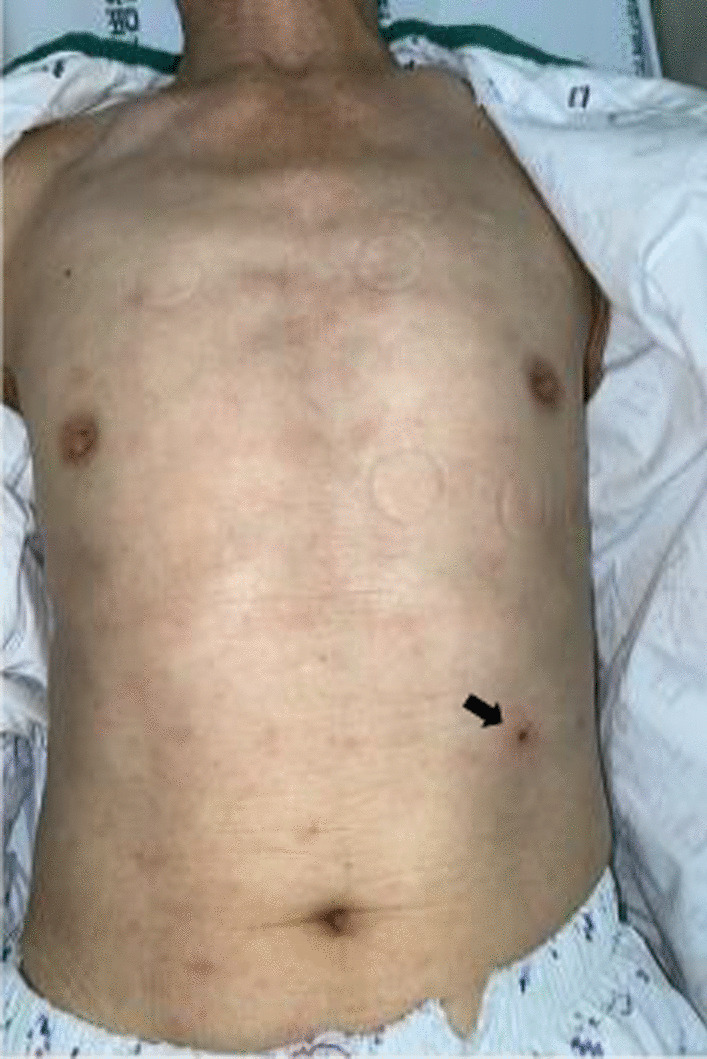
Eschar on the left upper abdomen quadrant (arrow)

A 78-year-old man presented to our emergency room with a 5-day history of fever and skin rash. His occupation was farming, and he predominantly grew peppers. On admission, his initial vital signs were a blood pressure of 130/80 mmHg, a heart rate of 88 beats per min, a respiratory rate of 16 breaths per min, and a temperature of 38.3 ℃. Physical examination by the emergency medical personnel revealed eschar on the left upper abdominal quadrant (Fig. 1). The patient was alert, and the neurological examination did not reveal any deficits. There was no cardiac murmur upon auscultation, and breath sounds were heard equally on both sides. His abdomen as a whole was not tender, and there was no history of trauma. His laboratory results were as follows: white blood cell count, 7.62 × 10^3^/μl (69.9% neutrophils); hemoglobin, 10.5 g/dL; platelet count, 74 × 10^3^/μl; alkaline phosphatase, 319 IU/L; gamma-glutamyl transpeptidase, 115 IU/L; total bilirubin, 0.74 mg/dL; aspartate transaminase (AST), 190 IU/L; alanine transferase (ALT), 113 IU/L; total protein, 5.1 g/dL; albumin, 3.2 g/dL; blood urea nitrogen (BUN), 43 mg/dL; creatinine, 1.76 mg/dL; lactate dehydrogenase (LDH), 824 IU/L; C-reactive protein, 189 mg/L; and procalcitonin, 1.03 ng/ml. The patient had not traveled abroad in the past year. Serology for the Epstein–Barr virus (EBV) indicated a previous EBV infection (negative VCA IgM, positive VCA IgG, negative early antigen antibody, and positive EBV nuclear antigen antibody). There was no evidence of malaria in the peripheral blood smear. The *O. tsutsugamushi* antibody titer using the indirect immunofluorescent antibody test was 1:160, and this increased to 1:640 in the follow-up test after 2 weeks. The patient was clinically diagnosed with scrub typhus and started taking oral doxycycline (100 mg twice daily) from the first day of admission.

**Fig. 2:**
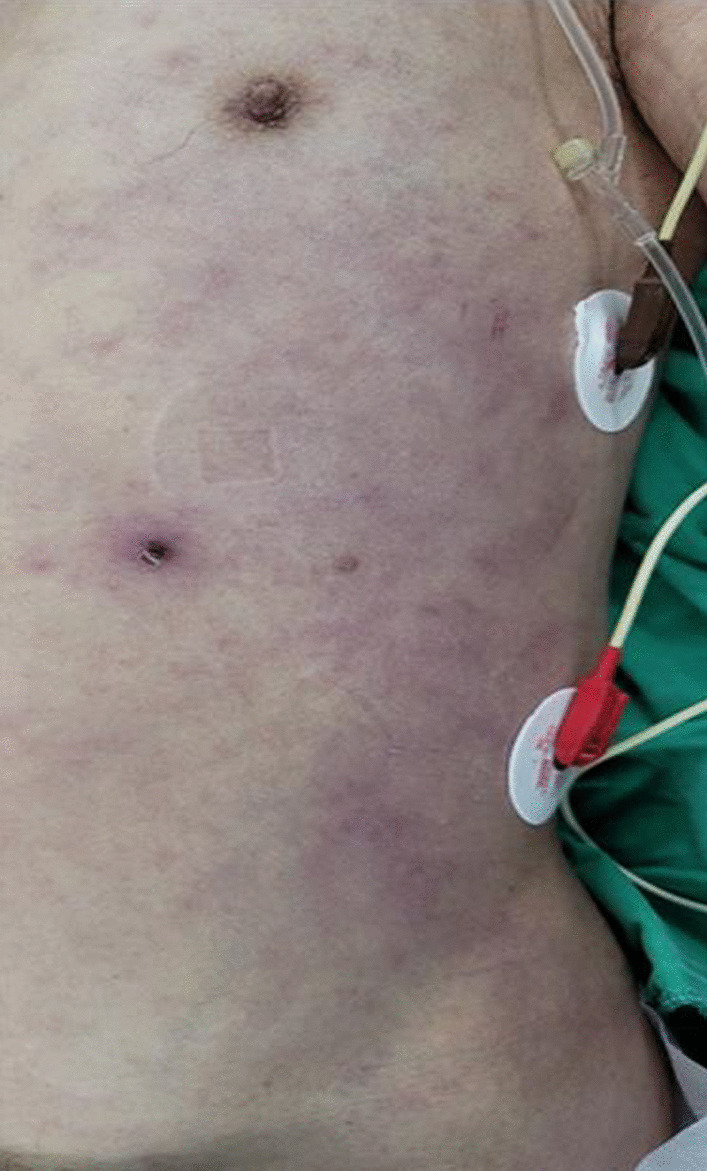
Grey Turner’s sign indicating bruising of the left flank

On Day 6 of hospitalization, the patient complained of sudden-onset left upper abdominal quadrant pain and showed mental changes. Patient was drowsy. He was not oriented, but was following commands. There was no cardiac murmur upon auscultation, and breath sounds were heard equally on both sides. During abdominal examination, direct tenderness was observed throughout the entire abdominal area. His blood pressure was 70/40 mmHg, heart rate 140 beats per min, respiratory rate 20 breaths per min, and temperature 36.8 ℃. He was transferred to the intensive care unit. The laboratory analysis performed immediately in the intensive care unit showed white blood cell count, 17.82 × 10^3^/μl (54.8% neutrophils); hemoglobin, 4.9 g/dL; and platelet count, 76 × 10^3^/μl. No signs associated with gastrointestinal bleeding, such as hematemesis, melena, or hematochezia, were observed. While preparing for an abdominal computed tomography (CT) to evaluate the patient’s pain, the patient went into ventricular tachycardia. Cardiopulmonary resuscitation (CPR) was done for 10 min and return of spontaneous circulation was achieved. Echocardiography performed after CPR showed near-normal left ventricular systolic function (ejection fraction 53%), left ventricular end-diastolic pressure (E/E’ ratio 13), mild resting pulmonary hypertension, and a small amount of pericardial effusion without hemodynamic significance. Abdominal CT was not done. Grey Turner's sign was suspected due to discoloration of the left flank on abdominal examination (Fig. 2), and portable ultrasonography revealed retroperitoneal bleeding, so an emergency exploratory laparotomy was undertaken. Based on the operative findings, hemoperitoneum due to splenic rupture was diagnosed and a splenectomy was performed.

**Fig. 3:**
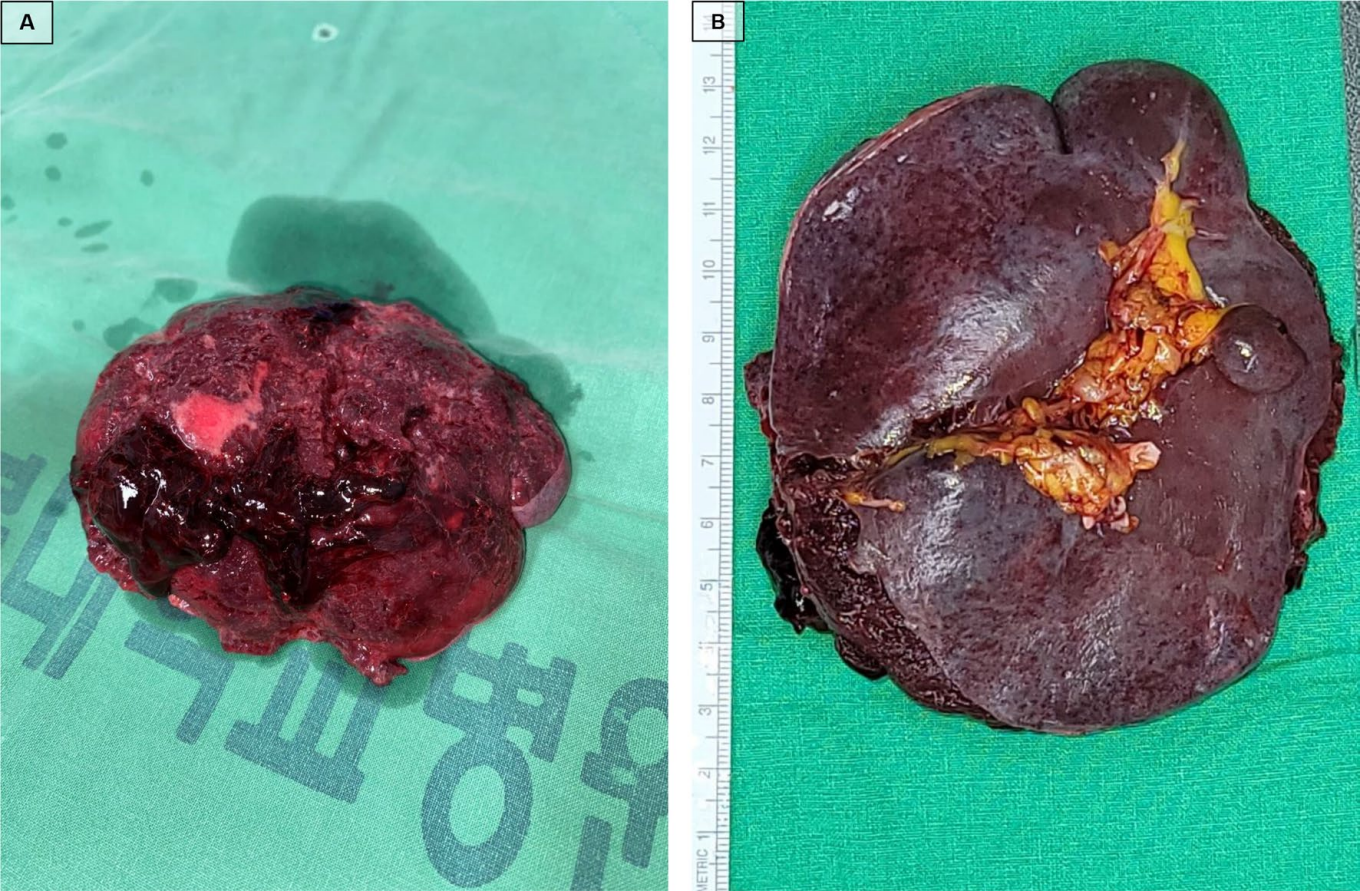
Macroscopic findings of a ruptured spleen. **A** The capsule of the dorsal surface of the spleen was torn, the spleen parenchyma was exposed, and coagulated blood clots were present along with bleeding in the ruptured area. **B** The hilum capsule of the spleen; no abnormalities were detected

**Fig. 4:**
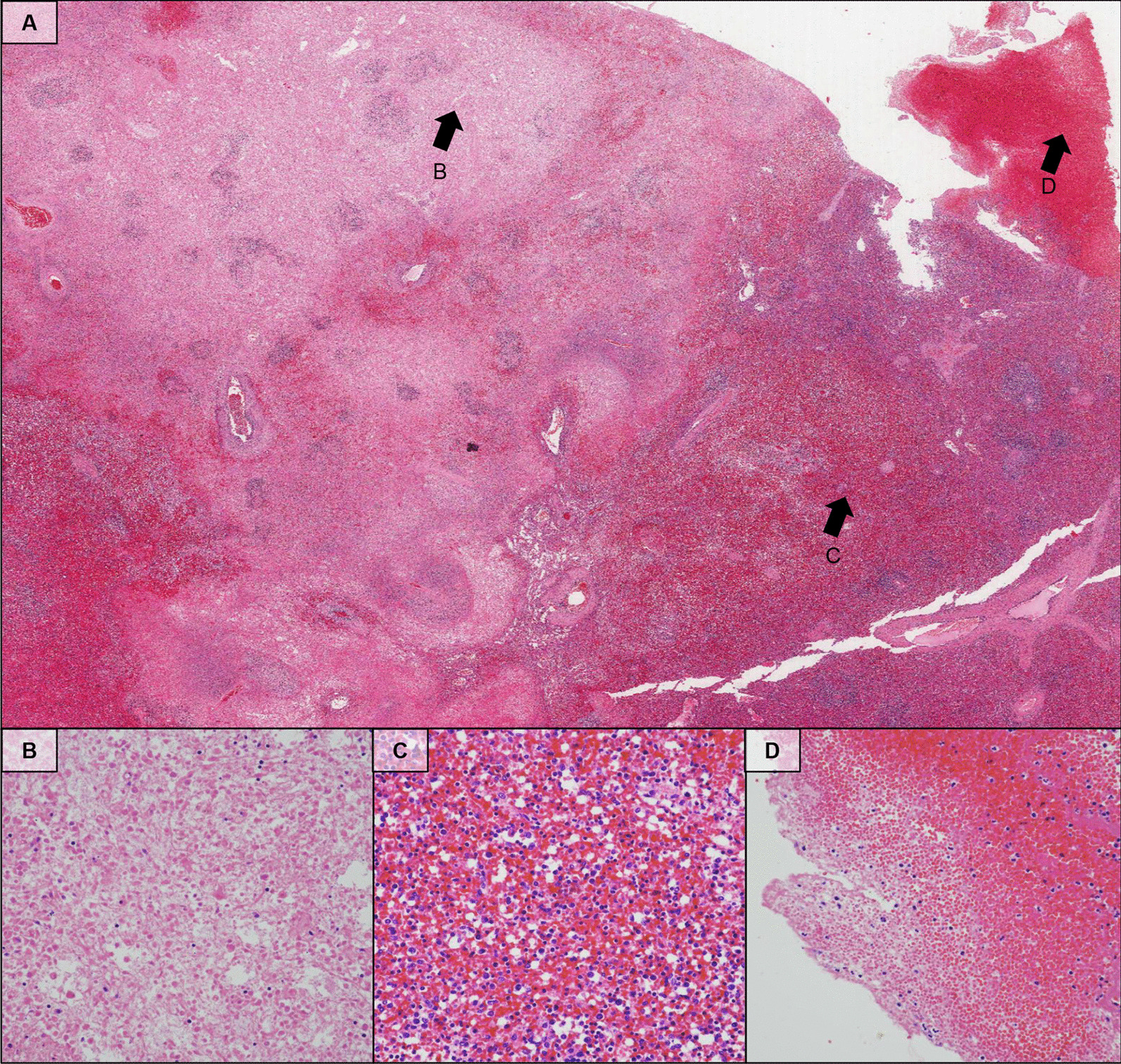
Histologic features of spleen. **A** Lower-power field of the spleen showing various types of injuries (H&E stain, original magnification: × 20). **B** High-power view of the faintly eosinophilic area in Figure A reveals infarction-type necrosis in the splenic tissue (H&E stain, arrow, original magnification: × 200). **C** Higher magnification on the hemorrhagic area of Figure A reveals the destruction of red pulp and congestion of red blood cells (H&E stain, arrow, original magnification: × 200). **D** High-power view of the splenic capsular region showing subcapsular hemorrhage (H&E stain, arrow, original magnification: × 200)

Figure 3 shows the macroscopic findings from the patient’s ruptured spleen. The capsule of the dorsal surface of the spleen was torn, the spleen parenchyma was exposed, and coagulated blood clots were present along with bleeding in the ruptured area (Fig. 3A). The hilum capsule of the spleen showed no abnormalities (Fig. 3B). Histopathology of the ruptured spleen revealed various types of injuries (Fig. 4A): infarction of the splenic tissue (Fig. 4B), red pulp of the spleen showing destruction of the splenic sinus with red blood cell congestion (Fig. 4C), and subcapsular hemorrhage (Fig. 4D).

After emergency surgery, oral doxycycline was terminated, and intravenous azithromycin (500 mg daily for 6 days) was administered along with intravenous vancomycin (750 mg q 72 h) and meropenem (500 mg q 24 h). Intravenous vancomycin and meropenem were administered for 14 days. During hospitalization, no bacteria were identified in the patient’s blood culture. There was no recurrence of ventricular tachycardia, and no arrhythmia associated with azithromycin was observed. However, renal failure with hemodialysis, persistent hyperbilirubinemia, and multiorgan failure occurred from the next day after surgery and persisted during hospitalization. He did not recover from these conditions and died on the fifty-sixth day of hospitalization.

## Discussion and conclusions

This study reports a patient with spontaneous splenic rupture associated with scrub typhus and, for the first time, presents the corresponding gross and histopathologic findings. Abdominopelvic involvement is not uncommon in patients with scrub typhus [7]. However, splenic rupture and splenic infarction as splenic complications associated with scrub typhus have been reported as rare [1, 7, 10, 11]. According to a systematic review of 845 patients with atraumatic splenic rupture, of the infectious disorders associated with splenic rupture, infectious mononucleosis is the most common, followed by malaria [12]. Another study reported the incidence of splenic rupture in infectious mononucleosis as 0.1% [13]. However, the estimated incidence of malaria-induced spleen rupture is 0.12%, and malaria is considered the most frequent cause of pathological splenic rupture worldwide, although the true incidence of pathological splenic rupture in natural vector-transmitted malaria is unknown [14, 15]. To date, the incidence of scrub typhus-related splenic rupture is also unknown. In three radiologic studies on scrub typhus, the frequency of splenic infarction was presented as 3.8% [7], 6.4% [11], and 16% [1]. Therefore, although there are limitations in assessing the incidence of scrub typhus-related splenic rupture, it is estimated to be lower than the risk of scrub typhus-related splenic infarction and splenic rupture in either infectious mononucleosis or malaria.

We reviewed the relevant literature discussing splenic rupture associated with scrub typhus by searching for “spleen rupture, scrub typhus” in MEDLINE/PubMed and KoreaMed (https://koreamed.org/). The two reported cases survived, but our patient died despite undergoing a splenectomy. Table 1 presents the clinical characteristics of the three cases. There were nine cases of scrub typhus-related splenic infarction reported in PubMed and KoreaMed [16–22]. Table 2 compares the clinical characteristics of the nine cases of splenic infarction and the three cases of splenic rupture. There were no statistically significant differences in the median time from symptom onset to splenic complications between the two groups (*P* = 0.402). All the patients with splenic infarction recovered with only medical treatment, but the splenic rupture cases were more likely to need surgical treatment, such as a splenectomy, because splenic rupture can be severe and life-threatening [12].

**Table 1:** The clinical feature, treatment, and outcome of patients with scrub typhus-related splenic rupture

	Case 1	Case 2	Present case
Age	74 years	75 years	78 years
Sex	Female	Male	Male
Abdominal pain	Yes (right side pain)	Yes (LUQ pain)	Yes (LUQ pain)
Blood pressure	109/93 mmHg	110/70 mmHg	70/40 mmHg
Heart rate	120 beats/min	88 beats/min	140 beats/min
Hemodynamic instability	Yes	No	Yes
Time from the onset of symptom to diagnosis of splenic rupture	5 days	14 days	11 days
Treatment			
Antibiotics, duration (day)	Chlorampenicol (PO, 1 day), doxycycline (PO, 6 days)	NA	Doxycycline (PO, 6 days) Azithromycin (IV, 6 days)
Medical treatment	Yes	Yes	Yes
Splenectomy	Done	Not done	Done
Outcome	Alive	Alive	Death

*NA* not available, *PO* per oral, *IV* intravenous, *LUQ* left upper quadrant

**Table 2:** Comparison of splenic infarction and splenic rupture in scrub typhus

	Splenic rupture (*n* = 3)	Splenic infarction (*n* = 9)
Abdominal pain or diffuse abdominal tenderness	3/3	8/9
Time from symptom onset to splenic complications, days, median (range)^a^	11 (5, 14)	7 (3, 15)
Only medical treatment	1/3	9/9
Splenectomy	2/3	0/9
Death	1/3	0/9

The figures in each cell refer to the number of subjects occurring among the total number of patients

^a^Mann–Whitney U test was used to assess time from symptom onset to splenic complications between 3 (3/3) patients with splenic rupture and 8 (8/9) patients with splenic infarction (*P* = 0.402)

To date, the mechanisms of scrub typhus-related splenic rupture have not been reported. Discussions regarding the concrete mechanisms of splenic rupture are limited because there are insufficient data about the underlying disease, as well as laboratory and pathological findings related to splenic rupture caused by scrub typhus. However, Kim et al.’s study suggested that intrasplenic pseudoaneurysm is one of the mechanisms [9]. Based on our study’s pathological findings, we hypothesize a sequence of events that could lead to extra-splenic hemorrhage: massive red blood cell congestion, which distorts and destroys the structure of the splenic sinus, and splenic infarction increase the volume of the red pulp and put pressure on the capsule, leading to capsular distension, rupture, and ultimately extra-splenic hemorrhage. This mechanism is similar to that of malaria-related splenic rupture [14]. Macroscopic investigations of splenomegaly found the capsule on the hilum side to be dilated, the capsule on the other side to bear a degloving injury, and the spleen parenchyma exposed to the abdominal cavity. Ischemic changes and capsular rupture meant the spleen appeared almost purple in color. This suggests that splenomegaly and capsular distention contribute to extra-splenic hemorrhage [14].

In summary, we report a patient with spontaneous scrub typhus-related splenic rupture. Clinicians should consider the possibility of splenic rupture in patients with scrub typhus who present with sudden-onset abdominal pain and unstable vital signs. We hypothesize that capsular rupture and extra-capsular hemorrhage result from splenomegaly and capsular distention, themselves caused by red blood cell congestion in the red pulp distorting and destroying the splenic sinus.

## Data Availability

The data supporting this study’s the findings are available from the corresponding author upon reasonable request.

## Abbreviations

CPR: Cardiopulmonary resuscitation
CT: Computed tomography
ROSC: Return of spontaneous circulation
LV: Left ventricle
EF: Ejection fraction
LVEDP: Left ventricular end-diastolic pressure
EAL: Early antigen
EBV: Epstein–Barr virus
EBNA: EBV nuclear antigen
